# Case report: a rare case of obsessive-compulsive disorder

**DOI:** 10.1192/j.eurpsy.2025.1713

**Published:** 2025-08-26

**Authors:** E. Ilgin, S. Göktaş, Ö. Gülerer, S. T. Tabur

**Affiliations:** 1 Psychiatry, Marmara University; 2 Psychology, Yeditepe University, Istanbul; 3 Psychology, Hasan Kalyoncu University, Ankara, Türkiye

## Abstract

**Introduction:**

Obsessive-compulsive disorder (OCD) is a chronic mental health condition characterized by intrusive thoughts (obsessions) and repetitive behaviors (compulsions), which significantly impair daily functioning and quality of life. While common obsessions often revolve around contamination, symmetry, or safety, rarer forms of OCD can involve highly specific and unusual fears.

**Objectives:**

The aim of this case analysis is to comprehensively examine the possible causes and treatment approaches of obsessive-compulsive disorder, focusing on the investigation of various obsessive conditions that are rarely observed in the literature.

**Methods:**

The patient’s history was thoroughly examined, and interviews conducted with the patient’s family were also included in the evaluation. Possible causes of the disorder (from a biopsychosocial perspective) and treatment approaches such as psychotherapy and pharmacotherapy were analyzed through a literature review. Additionally, the patient was assessed using the Yale-Brown Obsessive Compulsive Scale (YBOCS), Brown Beliefs Scale, Beck Anxiety Inventory, and Beck Depression Inventory.

**Results:**

The patient, N.C., is a 64-year-old woman whose general appearance is somewhat older than her age, with partially diminished self-care, having insight, divorced, and living with three of her six children. The patient experiences intense anger when any product containing sweets enters her home, including fruits. She feels discomfort even when using or hearing the word “sweet.” The patient insists that no family member brings any sweet-containing products into their home, leading to frequent arguments on the subject. She reports high levels of anxiety, difficulties in social relationships, and significant limitations in daily life activities. Her relatives have also observed and reported these challenges. The patient mentions feeling a sticky sensation on her hands when she sees sweets and experiences a compulsion to wash her hands when this sensation occurs. Her symptoms are consistent with the classical symptoms of obsessive-compulsive disorder, including obsessions (persistent thoughts about sweets and associated anger) and compulsions (the need to wash her hands). The diagnosis was made by Dr. Ece Ilgın.She has been started on fluvoxamine 50 mg/day, which will be titrated. She will return for a follow-up appointment at the clinic in one month.

**Conclusions:**

The symptoms of obsessive-compulsive disorder in patient N.C. include obsessions related to sweets, fruits, a sense of ‘stickiness’ that cannot be clearly identified, and avoidance obsessions linked to these. These symptoms significantly impact the patient’s quality of life and family relationships. It is believed that a combination of psychotherapy and pharmacotherapy will be effective in alleviating the patient’s symptoms and improving their quality of life.

**Disclosure of Interest:**

None Declared

